# Rare Duplication in the *RYR1* Gene Causing Malignant Hyperthermia and Clinical Variability

**DOI:** 10.3390/genes16111252

**Published:** 2025-10-24

**Authors:** Brandow W. Souza, Guilherme L. Yamamoto, Isabela A. Zogbi, Pamela V. Andrade, Joilson M. Santos, Leticia N. Feitosa, Acary S. B. Oliveira, Debora R. Bertola, Soledad Levano, Thierry Girard, Helga C. A. Silva, Mariz Vainzof

**Affiliations:** 1 Human Genome and Stem Cells Research Center, Institute of Biosciences, University of São Paulo, São Paulo 05508-090, SP, Brazil; brandow@usp.br (B.W.S.); guilherme.yamamoto@hc.fm.usp.br (G.L.Y.); isabela.zogbi@usp.br (I.A.Z.);; 2 Malignant Hyperthermia Center, Department of Anesthesiology, Pain and Intensive Care, Federal University of São Paulo, São Paulo 04039-032, SP, Brazil; pamela.vieira@unifesp.br (P.V.A.);; 3 Department of Biomedicine, University Hospital Basel, 4031 Basel, Switzerland; 4 Clinic for Anaesthesia, Intermediate Cre, Prehosptial Emergency Medicine and Pain Therapy, University Hospital Basel, University of Basel, 4031 Basel, Switzerland

**Keywords:** malignant hyperthermia, *RYR1*, duplication

## Abstract

**Background/Objectives**: Variants in the *RYR1* gene are associated mainly with Malignant Hyperthermia. Missense variants are largely the most common, while insertions and duplications account for less than 10%. We aimed to investigate the effect of a rare duplication in the *RYR1* gene with the variability of the Malignant Hyperthermia susceptibility phenotype. **Methods**: We used exome variant screening, in vitro contracture test, anatomopathological examination of the muscle biopsy, RT-qPCR analysis for *RYR1* relative expression. **Results**: We identified a family with two affected siblings carrying an insertion of 18 pair bases in exon 91 of the *RYR1* gene, resulting in an in-frame duplication of 6 amino acids (c.12835_12852 dupGAGGGCGCGGCGGGGCTC: 162 p.G4279_T4284insAAGLEG). This variant was found at a frequency of 0.0007% in gnomAD and was absent in 1200 Brazilian controls. First classified as a Variant of Uncertain Significance (VUS), with the molecular and physiological data from our family, we were able to reclassify it, reaching 5 points, which is still a VUS but borderline likely pathogenic. Muscle relative mRNA expression of *RYR1* in the two patients identified a ~50% reduction, suggesting a possible hypomorphic allele. **Conclusions**: The pathomechanisms of *RYR1* gene variants in Malignant Hyperthermia are mainly associated with gain-of-function mechanisms, but small insertions can often lead to loss of function or improper folding protein. This study adds evidence to the possibility that duplication in this region can cause structural defects and a more severe phenotype in the patients.

## 1. Introduction

Malignant Hyperthermia (MH) is a life-threatening pharmacogenetic disorder of skeletal muscle, classically inherited in an autosomal dominant manner. It is triggered primarily by exposure to certain volatile anesthetic agents (e.g., halothane, isoflurane, desflurane, sevoflurane) and the depolarizing neuromuscular blockers succinylcholine. In rare instances, episodes may also be precipitated by intense physical exertion or elevated environmental temperatures. The condition is characterized by a hypermetabolic crisis, which can manifest clinically as hypercapnia, tachypnea, tachycardia, hyperthermia, metabolic acidosis, hyperkalemia, muscle rigidity, and rhabdomyolysis. The estimated incidence of MH reactions ranges from 1 in 10,000 to 1 in 250,000 anesthetic procedures, depending on population and diagnostic criteria [1].

In addition to MH, *RYR1* mutations have been implicated in a spectrum of congenital myopathies, including central core disease (CCD), multiminicore disease, King-Denborough syndrome, centronuclear myopathy, and congenital fiber-type disproportion. The clinical presentation among carriers of *RYR1* variants is highly variable, and the genetic landscape is marked by high allelic heterogeneity, incomplete penetrance, and variable expressivity. The primary gene implicated in MH susceptibility is *RYR1*, which accounts for approximately 75% of genetically confirmed cases. Pathogenic variants in this gene result in dysregulated calcium release from the sarcoplasmic reticulum, leading to sustained muscle contraction and metabolic crisis [2]. In fact, around three out of four families at risk for MH have been found to carry pathogenic mutations in the *RYR1* gene.

*RYR1* is located on chromosome 19q13.1 and encodes the ryanodine receptor type 1 (*RYR1*), a calcium release channel embedded in the terminal cisternae of the sarcoplasmic reticulum in skeletal muscle fibers. This receptor plays a central role in excitation–contraction coupling by mediating the release of Ca^2+^ into the cytoplasm in response to depolarization signals [2].

The *RYR1* gene comprises 106 exons and encodes a large protein with 5038 amino acids [3]. Due to its extensive size and complexity, *RYR1* has historically been a challenging gene to study. For many years, research efforts were mainly concentrated on three domains (N-terminal, central and C-terminal) well-established mutation hotspots. However, with the advent of Next-Generation Sequencing (NGS) technologies, it has become possible to analyze the entire gene. As a result, numerous novel variants have been identified outside of the previously known hotspots.

To date, more than 1000 distinct *RYR1* variants have been reported (HGMD; https://www.hgmd.cf.ac.uk/ (accessed on 13 February 2025) and LOVD; https://databases.lovd.nl/shared/genes/ *RYR1*, (accessed on 13 February 2025)), yet only 72 have been classified as diagnostic mutations by the European Malignant Hyperthermia Group (EMHG; https://www.emhg.org/diagnostic-mutations (accessed on 13 February 2025)). The vast majority of these are missense mutations, while insertions, deletions, and duplications are relatively uncommon, representing less than 10% of all known pathogenic variants in *RYR1* gene. Therefore, studying patients who carry CNVs is essential to better understand the impact of this type of mutation on *RYR1* channel function and its correlation with clinical phenotype.

In a recent molecular study of 61 MH Brazilian families [4], we identified one family with a patient carrying a duplication of 18 pb in the *RYR1* gene. The study of the whole family can give new insights into this type of variant, expanding our understanding of *RYR1*-related pathogenic mechanisms.

This study aimed to investigate the effect of a rare duplication in the *RYR1* gene in the variability of the phenotype in Malignant Hyperthermia susceptibility, thereby contributing to the elucidation of its pathogenicity.

## 2. Materials and Methods

**Figure 1:**
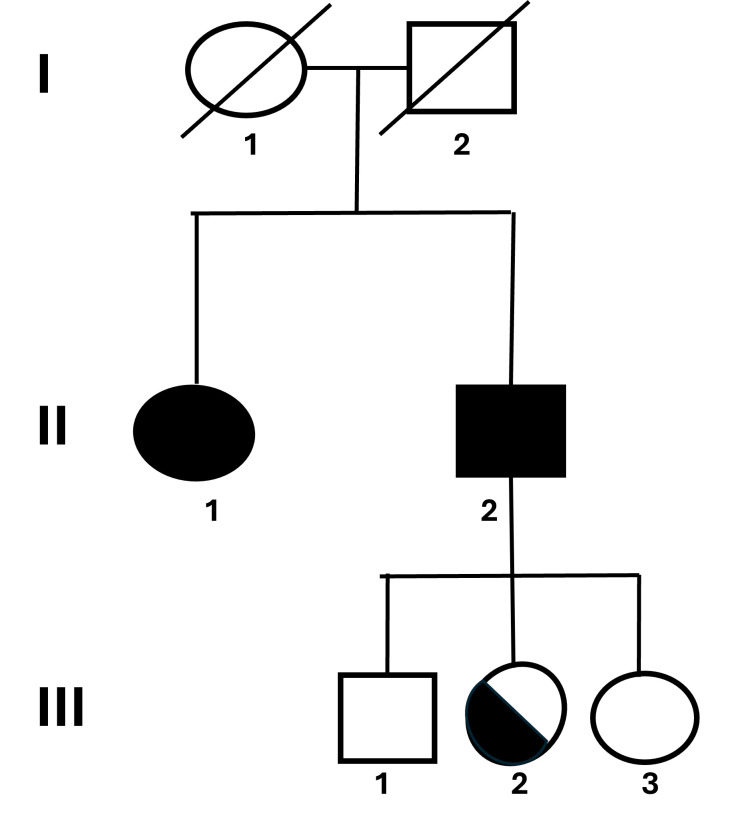
Pedigree and clinical overview of the family carrying the *RYR1* duplication. Pedigree showing the inheritance pattern of the *RYR1* exon 91 duplication (c.12835_12852dupGAGGGCGCGGCGGGGCTC; p.G4279_T4284insAAGLEG) in a family with Malignant Hyperthermia (MH). Two affected siblings (II.1 and II.2) presented positive IVCT results and variable clinical manifestations including muscle rigidity and hyperthermia upon anesthesia exposure. The asymptomatic carrier (III.2) inherited the same duplication, whereas the remaining relatives were negative. Squares represent males and circles represent females; filled symbols indicate affected individuals, and half-filled symbols indicate asymptomatic carriers.

Five members from a family with history of malignant hyperthermia were evaluated (Figure 1). The Institutional Research Ethics Committee approved this research, and all participants provided their written informed consent.

### 2.1. Molecular Analysis

Genomic DNA was extracted from peripheral blood lymphocytes using standard protocols. Initially, both affected siblings underwent sequencing with the Illumina TruSight One Expanded panel (Illumina, San Diego, CA, USA), which targets over 6700 genes and clinically relevant exonic regions. Several years later, whole-exome sequencing (WES) was performed in one of the siblings. Additionally, WES was also conducted on his three children. For all exome analyses, libraries were prepared using the SureSelectQXT V6 Reagent Kit (Agilent, Santa Clara, CA, USA), and sequencing was carried out on an Illumina HiSeq2500 platform. The resulting FASTQ files were aligned to the human reference genome (GRCh38/hg38) using BWA-MEM-software to aligner to a reference genome, last version, SAM files were converted to BAM and PCR duplicates were marked with Picard Tools (v2.18.7). Variant calling followed GATK Best Practices and was performed with the UnifiedGenotyper module from GATK (v3.7). Variant annotation was carried out using ANNOVAR (7 Jun 2020). Both the annotated VCF and BAM files were manually reviewed to identify potentially pathogenic variants.

Variant filtering was performed by comparing allele frequencies across several control population databases, including 1000 Genomes, Exome Sequencing Project (ESP6500, Washington University), NIH, gnomAD, and the Online Archive of Brazilian Mutations (AbraOM—http://www.abraom.ib.usp.br/ (accessed on 13 February 2025)). Rare variants identified in the *RYR1* gene (OMIM#180901) were prioritized and further evaluated using in silico prediction tools. Previously reported pathogenic variants were cross-referenced in mutation databases such as HGMD, LOVD, and ClinVar. In silico analysis of the potential pathogenicity of novel or rare variants was conducted using multiple predictive algorithms, including MutationTaster, PredictSNP1, CADD, DANN, FATHMM, FunSeq2, GWAVA, VEP, SIFT, PolyPhen-2, and Human Splicing Finder 3.0.

For the classification of this duplication, we applied the guidelines established by the Association for Clinical Genomic Science (ACGS) [5], which integrates ACMG [6] criteria with additional frameworks, including those developed by ClinGen, to support and enhance variant classification.

### 2.2. RT-qPCR Analysis

To assess gene expression, total RNA was isolated from muscle biopsy samples obtained from both patients and control individuals. RNA extraction was performed using the TRIzol™ Reagent (Invitrogen, Carlsbad, CA, USA), following the protocol provided by the manufacturer. RNA concentration and purity were evaluated using a NanoDrop spectrophotometer (Thermo Fischer Scientific, Waltham, MA, USA), and samples were subsequently stored at −70 °C. The cDNA was synthesized from 1 µg of total RNA using the SuperScript™ VILO™ Master Mix kit (Invitrogen). The reverse transcription reaction was conducted in a thermocycler for 10 min at 25 °C, 60 min at 42 °C, and 5 min at 85 °C. The resulting cDNA was diluted prior to use in further analyses. For quantification of *RYR1* gene expression, 10 µg/mL of cDNA was used. The *GAPDH* gene expression values were used as endogenous reference for normalization. Quantitative real-time PCR was performed using PowerUp SYBR Green Master Mix (Applied Biosystems, Waltham, MA, USA) on a QuantStudio™ 5 Real-Time PCR System (Applied Biosystems) following the thermocycling program: 95 °C for 10 min, followed by 40 cycles of 95 °C for 15 s and 60 °C for 1 min. Relative expression levels were calculated using the 2^−ΔΔCt^ method.

Primers were designed according to parameters required for RT-qPCR. The primer sequences were as follows:

- *RYR1* forward: 5′-ACGGAGAGAAGGTCATGGCG-3′ and reverse: 5′-CCGCAGGAGGTAGTCCACAG-3′;
- *GAPDH* forward: 5′-TGCACCACCAACTGCTTAGC-3′ and reverse: 5′-GGCATGGACTGTGGTCATGA-3′.

### 2.3. IVCT

In vitro contracture test (IVCT) was performed according to the EMHG protocol. The positive IVCT result was reported as MHShc when contractures developed both to halothane and caffeine, MHSh when contractures developed only to halothane, and MHSc when contractures developed only to caffeine.

### 2.4. Anatomopathologic Study

Muscle strips obtained from vastus lateralis under regional anesthesia were used for the stains and histochemistry studies (haematoxylin-eosin, Gomori trichrome, Sudan, periodic acid Schiff, nicotinamide adenine dinucleotide dehydrogenase (NADH), succinate dehydrogenase (SDH), cytochrome c oxidase (COX), and myofibrillar adenosine triphosphatase (ATPase) with alkaline (pH 9.4) and acid (pH 4.3) preincubation).

## 3. Results

### Description of the Family

The propositus of the family was an 18-year-old female presenting an atypical reaction to anesthesia. At the age of 5 years old, the patient underwent a surgical correction of an elbow fracture and presented hyperthermia and muscle rigidity during the anesthesia, with complete reversal of symptoms after interruption of the procedure and administration of dantrolene. The in vitro contracture test (IVCT), according to the EMHG criteria, was performed, displaying a positive reaction for both halothane and caffeine (MHShc), with contracture of 0.64 g in response to 2% halothane and 0.32 g to 2 mM caffeine. Subsequently, her brother opted to also be evaluated. At the age of 25 years old, he was subjected to a muscle biopsy and IVCT, which also showed positive reaction -MHShc—contracture of 1.12 g in response to 2% halothane and 0.32 g to 2 mM caffeine—supporting the diagnosis of MH. The pedigree of the family is shown in Figure 1.

**Figure 2:**
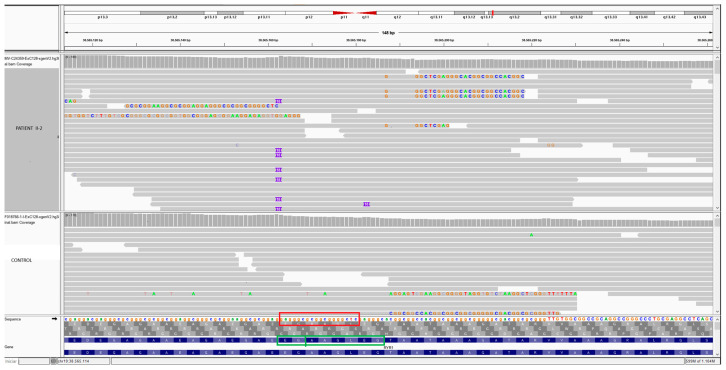
IGV image showing a comparison between a control and patient II-2 with the duplication in exon 91 of *RYR1*. In the bottom: red square showing the nucleotide sequences of 18-bp of the duplication (GAGGGCGCGGCGGGGCTC). The green square showing the aminoacids sequence. The aminoacids EG can be localized in both sides of the sequence. HGVS variant notation recommends right alignment in relation to transcript and therefore AAGLEG.

Molecular genetic analysis was performed using NGS methodology in both sibs and identified the variant (NM_000540.3) c.12835_12852 dupGAGGGCGCGGCGGGGCTC: p.G4279_T4284insAAGLEG in exon 91 of the *RYR1* gene that result in a duplication of 6 aa (AAGLEG) in the position 4284 of the polypeptide. The aminoacids EG can be localized in both sides of the sequence (Figure 2). HGVS variant notation recommends right alignment in relation to transcript and therefore AAGLEG.

This insertion was found at a frequency of 0.0007% in gnomAD and was not present in normal 1200 Brazilian controls (AbraOM—Brazilian Genomic Variants). This variant has been previously reported in ClinVar as VUS, (rs1159068582), with low frequency in population databases but with no information in the literature in individuals affected with *RYR1*-related conditions. Also, experimental studies and prediction algorithms were not available or evaluated, and the functional significance of this variant is currently unknown. With the molecular and physiological data from our family, we were able reclassify the variant with the following criteria: PM2_sup, PM4, PS4_sup, PS3_sup, reaching 5 points which is still a VUS but borderline likely pathogenic (up to 6 points would be likely pathogenic).

A detailed clinical examination in patient one (II.1) showed in addition to the atypical reaction to anesthesia with positive IVCT, clubfoot, exercise intolerance, high-arched palate, proximal paresis of all the four limbs and hyporeflexia. Histopathological analysis of the muscle revealed increased variability in fiber size, hypertrophic and atrophic fibers, type I fiber predominance (63% and atrophy. She presented normal creatine kinase (CK). Patient II.2 also exhibited positive IVCT but CK levels were reaching approximately 20 times the upper limit of normal after physical effort. Clinical evaluation described clubfoot, ptosis, kyphosis and high-arched palate. Histopathological findings in his muscle biopsy were normal.

Considering the family history, the 3 children from patient 2 were invited to be studied. The 20-year-old son (patient III.1), the 18-year-old daughter (patient III.2) and the 13-year-old daughter (patient III.3) were all with no evident clinical signals. Only the daughter III-2 presented the duplication in the molecular analysis.

**Figure 3:**
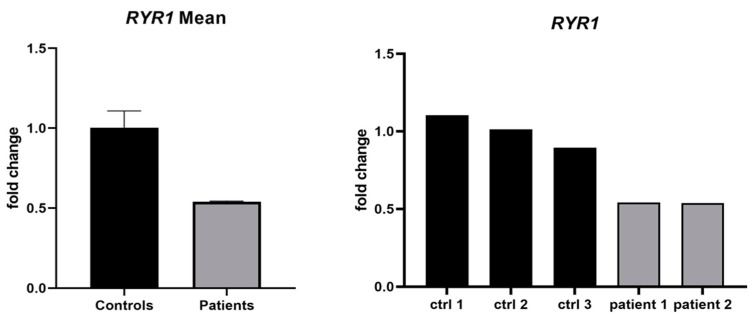
Relative expression of *RYR1* mRNA in muscle biopsy samples. Bar plot showing the relative expression of *RYR1* mRNA in muscle biopsies from two affected individuals (II.1 and II.2) compared with 3 control samples. Expression levels were quantified using RT-qPCR with *GAPDH* as the endogenous control, and the 2^–ΔΔCt^ method was applied for normalization. A reduction of approximately 50% in *RYR1* transcript levels was observed in both patients, suggesting a potential hypomorphic effect of the duplicated allele.

We studied the relative expression of *RYR1* in mRNA extracted from the muscle biopsies from patients II.1 and II.2 and identified a ~50% reduction in its expression. Although not statistically significant, it could suggest the presence of a hypomorphic allele (Figure 3).

## 4. Discussion

The duplication identified in this family, according to ClinVar (https://www.ncbi.nlm.nih.gov/clinvar/ (accessed on 13 February 2025)), has been reported twice as VUS, once in 2020 and again in 2024, with neither submission providing additional evidence supporting pathogenicity. According to the gnomAD database v4.10 (https://gnomad.broadinstitute.org/ (accessed on 13 February 2025)), the total allele frequency is extremely low, at 0.00067% (10 out of 1,471,840 alleles), and it was observed only in heterozygous state. It was most frequent in the East Asian population (0.0027%), followed by the African/African American (0.0014%) and European (0.0007%) populations. The more detailed clinical evaluation revealed that the two patients from this family presented some dimorphisms and clinical signals compatible with congenital myopathy, suggesting a possible deleterious role of the duplication in the function of the *RYR1* channel. The complementary molecular analysis of the 3 children from patients II.2 identified this variant only in one daughter (III-2) still with no dysmorphic characteristics.

Consulting the frequency of CNV associated with the *RYR1* gene in the HGMD bank, seven duplication or insertion variants have been identified associated with the phenotype of MH or *RYR1*-related diseases. Two of these correspond to chromosomal-level duplications involving multiple exons [7,8] and were not included in the present analysis.

Among the remaining five variants, three were duplications or insertions in exons 39, 91, and 102 and were found in compound heterozygosity, each segregating with another pathogenic or likely pathogenic variant in *RYR1* [9,10,11]. These combinations complicate the attribution of phenotypic effects to a single duplication variant and suggest possible additive or modifying effects. On the other hand, the remaining two monoallelic variants resulted in the phenotype of CCD myopathy, with the variants located in the C-terminal region. One case was described by Hunter et al. [12] and involves a 21-base pair in-frame duplication in exon 102 (c.14758_14778dupACCTTCTTCTTCTTCGTCATC; p.Thr4920_Ile4926dup), observed in both a father and son. The duplication is located within the final hydrophobic transmembrane domain of *RYR1*, a known pathogenic hotspot. This variant is associated with congenital myopathy and is thought to have arisen de novo in the father. Functional predictions suggest that the addition of seven amino acids in this critical domain may alter *RYR1* channel folding and function, possibly exerting a dominant-negative effect by interfering with *RYR1* tetramer assembly. The second case, reported by Erendzhinova et al. [13], involves a 27-base pair duplication in exon 101 (c.14545_14571dupCGTCTACCTGTACACCGTGGTGGCCTTC), identified in two maternal half-brothers diagnosed with CCD. Interestingly, one sibling presented with a mild phenotype, while the other had a severe form of disease. The mother tested negative for the variant, suggesting the presence of germline mosaicism. This duplication is also located within the C-terminal region, which is strongly associated with CCD severity.

As to region codified by exon 91, an in-frame duplication of 9 base pairs in exon 91 of *RYR1* (p.T4288_A4290dup) has previously been reported by different authors in unrelated families presenting phenotypes associated with *RYR1*-related diseases. It is also important to notice that in most of these cases, individuals with phenotypes related to *RYR1* disorders carried additional other variants in *RYR1*. Patients exhibited MH susceptibility, exertional myalgia and rhabdomyolysis [14,15]. According to gnomAD, this recurrent variant has a higher allele frequency in individuals of African ancestry (2.6%) if compared to total allele frequency (0.15%). Additionally, ClinVar indicates conflicting classifications of pathogenicity.

Exon 91 of the *RYR1* gene is a region with challenging sequence features, and difficult to study. According to the sequence available in Ensembl, this exon is composed of highly repetitive base pairs and exhibits a strong GC content, with 30.1% cytosine (C) and 45.6% guanine (G). The repetitive nature and elevated GC content of this exon can complicate both amplification and sequencing processes, potentially leading to reduced coverage, misalignment, or difficulty in detection and variant calling. For this reason, and to ensure a clear distinction between our variant and the variants p.T4288_A4290dup, we analyzed and compared the sequences from our study and the reported in case S2 by Levano et al. [15], confirming that the variants are molecularly different and cannot be classified as equivalent.

Our duplication introduces six additional amino acids near the beginning of the transmembrane domain of *RYR1*, a region known to play a crucial role in channel gating and inter-domain coupling. Given the high structural conservation of this region, even small in frame insertions could alter local folding or the spatial arrangement of neighboring helices, potentially affecting calcium release dynamics or Dihydropyridine receptor–*RYR1* coupling [16,17]. Predictive structure studies could elucidate the effect of this larger duplication in a region of the *RYR1* protein, just close to the transmembrane domains of the protein. A structural rearrangement could complicate or impair the insertion of the channel in the membrane and harm its function.

The relative expression of *RYR1* in muscle biopsies from the two affected individuals showed an approximately 50% reduction compared with control samples. Although this decrease may suggest a hypomorphic effect of the duplicated allele, the difference was not statistically significant (*p* = 0.20). Given the extremely small sample size (n = 2), this observation should be considered preliminary. Nevertheless, the downward trend remains biologically relevant and could reflect reduced mRNA stability or altered transcriptional regulation associated with the duplication. Future studies including additional affected and unaffected carriers will be necessary to confirm this tendency.

## 5. Conclusions

We identified a rare in-frame duplication in exon 91 of the *RYR1* gene associated with variability in the phenotype of MH susceptibility, characterized by dysmorphisms and mild myopathy. The duplication results in the insertion of six amino acids within the C-terminal region of *RYR1*, near the transmembrane domain that plays a critical role in calcium channel gating.

The two affected siblings carrying this variant exhibited a trend toward ~50% reduced *RYR1* mRNA expression, and although preliminary, could indicate a potential hypomorphic effect.

Altogether, these findings suggest that the exon 91 duplication may contribute to structural alterations in the C-terminal region, potentially impacting calcium homeostasis and predisposing to malignant hyperthermia. However, identifying additional affected individuals carrying this variant will be essential to promote its classification as likely pathogenic. Further investigations using calcium functional assays will be important to clarify the mechanism by which this duplication contributes to the MH phenotype.

## Data Availability

The original contributions presented in this study are included in the article. Further inquiries can be directed to the corresponding author Mariz Vainzof, mvainzof@usp.br.
